# Metabolic alteration of *Catharanthus roseus* cell suspension cultures overexpressing *geraniol synthase* in the plastids or cytosol

**DOI:** 10.1007/s11240-018-1398-5

**Published:** 2018-02-24

**Authors:** Mohd Zuwairi Saiman, Karel Miettinen, Natali Rianika Mustafa, Young Hae Choi, Robert Verpoorte, Anna Elisabeth Schulte

**Affiliations:** 1 Institute of Biology, Leiden University, 2300 RA Leiden, The Netherlands; 2 Institute of Biological Sciences, Faculty of Science, University of Malaya, 50603 Kuala Lumpur, Malaysia; 3 Centre for Research in Biotechnology for Agriculture (CEBAR), University of Malaya, 50603 Kuala Lumpur, Malaysia; 4 ExPlant Technologies B.V., Galileiweg 8, 2333 BD Leiden, The Netherlands

**Keywords:** *Catharanthus roseus*, Geraniol synthase, Metabolic engineering, Metabolomics, Plant cell culture, Alkaloids

## Abstract

Previous studies showed that geraniol could be an upstream limiting factor in the monoterpenoid pathway towards the production of terpenoid indole alkaloid (TIA) in *Catharanthus roseus* cells and hairy root cultures. This shortage in precursor availability could be due to (1) limited expression of the plastidial *geraniol synthase* resulted in a low activity of the enzyme to catalyze the conversion of geranyl diphosphate to geraniol; or (2) the limitation of geraniol transport from plastids to cytosol. Therefore, in this study, *C. roseus*’s *geraniol synthase* (*CrGES*) gene was overexpressed in either plastids or cytosol of a non-TIA producing *C. roseus* cell line. The expression of *CrGES* in the plastids or cytosol was confirmed and the constitutive transformation lines were successfully established. A targeted metabolite analysis using HPLC shows that the transformed cell lines did not produce TIA or iridoid precursors unless elicited with jasmonic acid, as their parent cell line. This indicates a requirement for expression of additional, inducible pathway genes to reach production of TIA in this cell line. Interestingly, further analysis using NMR-based metabolomics reveals that the overexpression of *CrGES* impacts primary metabolism differently if expressed in the plastids or cytosol. The levels of valine, leucine, and some metabolites derived from the shikimate pathway, i.e. phenylalanine and tyrosine were significantly higher in the plastidial- but lower in the cytosolic-*CrGES* overexpressing cell lines. This result shows that overexpression of *CrGES* in the plastids or cytosol caused alteration of primary metabolism that associated to the plant cell growth and development. A comprehensive omics analysis is necessary to reveal the full effect of metabolic engineering.

**Electronic supplementary material:**

The online version of this article (10.1007/s11240-018-1398-5) contains supplementary material, which is available to authorized users.

## Introduction

*Catharanthus roseus* (Madagascar periwinkle) is a medicinal plant which produces bioactive terpenoid indole alkaloids (TIA) such as the antihypertensive drugs ajmalicine and serpentine, as well as the antineoplastic agents, vinblastine and vincristine. However, most TIA especially the dimeric TIA are produced at low levels in the plant, difficult to extract or isolate, and unfeasible for total chemical synthesis due to their complex structures, which explain the high market prices of TIA (Pan et al. 2016).

Biotechnological approaches using in vitro cell and tissue cultures of *C. roseus* have been developed as an alternative source of TIA. However, a high producing cell line has not been obtained despite all efforts in the optimization of growing and production conditions. Although the mass cultivation of *C. roseus* cells is feasible in a large-scale bioreactor, the cost of production of alkaloids is too high for commercialization (Verpoorte et al. 2000). Metabolic engineering by overexpressing the biosynthetic genes of the limiting pathway or suppressing the flux of competing pathways are promising approaches to improve the production of TIA in *C. roseus* cell cultures (Verpoorte et al. 2000; Zhao and Verpoorte 2007).

**Fig. 1:**
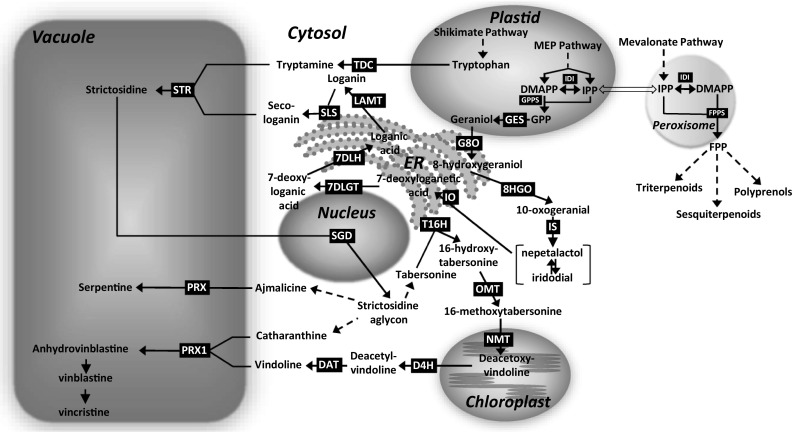
Scheme of the subcellular localization of enzymes in TIA pathway of *Catharanthus roseus*. Dashed arrows indicate multiple steps. Open arrow represents cross-flow between plastidial MEP and cytosolic/peroxisomal mevalonate pathway. *ER* endoplasmatic reticulum, *DMAPP* dimethylallyl diphosphate, *IPP* isopentenyl diphosphate, *IDI* IPP isomerase, *GPP* geranyl diphosphate, *GPPS* GPP synthase, *FPP* farnesyl diphosphate, *FPPS* FPP synthase, *GES* geraniol synthase, *G8O* geraniol 8-oxidase, *8-HGO* 8-hydroxygeraniol oxidoreductase, *IS* iridoid synthase, *IO* iridoid oxidase, *7DLGT* 7-deoxyloganetic acid glucosyl transferase, *7DLH* 7-deoxyloganic acid hydroxylase, *LAMT* loganic acid *O*-methyltransferase, *SLS* secologanin synthase, *STR* strictosidine synthase, *SGD* strictosidine *β*-d-glucosidase, *T16H* tabersonine 16-hydroxylase, *OMT* 16-hydoxytabersonine 16-*O*-methyltransferase, *NMT N*-methyltransferase, *D4H* desacetoxyvindoline 4-hydroxylase, *DAT* deacetylvindoline 4-*O*-acetyltransferase, *PRX* peroxidase, *PRX1* peroxidase 1, *TDC* tryptophan decarboxylase

Metabolic engineering requires knowledge on the biosynthesis pathway of the products of interest, the subcellular compartmentation of specific steps in the pathway, and the transport of the intermediates between intracellular compartments and between different cell types. The biosynthesis of TIA in *C. roseus* is a complex metabolic pathway involving different subcellular compartments including plastids, cytosol, nucleus, endoplasmic reticulum (ER) and vacuole (Fig. 1).

TIA biosynthesis requires two precursors from two different biosynthetic routes, i.e. tryptamine from the shikimate/tryptophan pathway and secologanin from the iridoid/methyl erythritole phosphate (MEP) pathway (Pan et al. 2016). The MEP pathway leading to geraniol is localized in the plastids. Geraniol synthase (GES) catalyzes the conversion of geranyl diphosphate (GPP) to geraniol in the plastid stroma and stromules (Simkin et al. 2013). Geraniol is then transported to the ER, where the next enzyme geraniol 8-oxidase (G8O) or known as geraniol 10-hydroxylase (G10H) is localized (Guirimand et al. 2009). A series of enzymes for conversion of 8-hydroxygeraniol (or known as 10-hydroxygeraniol) to loganic acid is shown to be localized in the cytosol (iridoid synthase [IS]) (Geu-Flores et al. 2012), both the cytosol and nucleus (8-hydroxygeraniol oxidoreductase [8HGO] and 7-deoxyloganic acid glucosyl transferase [7DLGT]), and the ER (iridoid oxidase [IO] and 7-deoxyloganic acid hydroxylase [7DLH]) (Miettinen et al. 2014). Loganic acid methyl transferase (LAMT) forming loganin is localized in the cytosol, whereas secologanin synthase (SLS) which catalyzed the formation of secologanin is anchored to the cytosolic face of the ER membranes (Guirimand et al. 2011a).

The shikimate pathway leading to tryptophan is also derived from the plastids and it has to be exported to the cytosol, where tryptophan decarboxylase (TDC) is mainly operated to yield tryptamine (De Luca and Cutler 1987). Strictosidine synthase (STR) was shown to be localized in the vacuole, thus tryptamine and secologanin from the MEP pathway need to be transported to the vacuole to produce strictosidine. Subsequently, strictosidine is transported out of the vacuole to be deglucosylated by strictosidine-*β*-d-glucosidase (SGD) which is associated with the nucleus (Guirimand et al. 2010). Further TIA biosynthesis towards vinblastine and vincristine involves a series of enzymatic reaction localized in the ER (tabersonine 16-hydoxylase [T16]), cytosol (16-hydroxytabersonine 16-*O*-methyltransferase [OMT], desacetoxyvindoline 4hydroxylase [D4H], and deacetylvindoline 4-*O*-acetyltransferase [DAT]), thylakoid membrane of chloroplasts (*N*-methyltransferase [NMT]) and vacuole (peroxidase [PRX1]) (De Luca and Cutler 1987; Costa et al. 2008; Guirimand et al. 2011b).

Among the two precursor pathways, the iridoid pathway is considered a major rate-limiting factor for TIA production in *C. roseus* cell cultures (Zhao and Verpoorte 2007; Pan et al. 2016). The iridoid precursors of the TIA derive from 8-hydroxygeraniol (also known as 10-hydroxygeraniol) which is formed upon hydroxylation of geraniol generated from GPP. GPP is a condensation product of the basic isoprene units, isopentenyl diphosphate (IPP) and dimethylallyl diphosphate (DMAPP). In plants, IPP and DMAPP are produced via two different metabolic pathways each leading to a distinct set of terpenoid derivatives (Fig. 1). These two pathways closely interact but they are separated on subcellular level, i.e., the cytosolic/peroxisomal mevalonate pathway (Sapir-Mir et al. 2008; Thabet et al. 2011) and the plastidial MEP pathway (Rohmer 1999). The iridoid/terpenoid moiety of TIA derives from GPP produced via the MEP pathway (Contin et al. 1998). This intermediate is primarily produced by the plastidial enzyme geranyl diphosphate synthase (GPPS), but can also be released as an intermediate during the formation of farnesyl diphosphate (FPP) catalyzed by FPP synthase (FPPS), which is localized in the peroxisome/cytosol (Thabet et al. 2011). Although the GPP production in the mevalonate pathway remains unclear, a few studies indicate that a low GPP pool was available in the cytosol for the synthesis of limonene in transgenic *Nicotiana tabacum* (Wu et al. 2006) and geraniol in transgenic *Nicotiana benthamiana* (Dong et al. 2013).

Single or multiple genes encoding the biosynthesis enzymes of the TIA pathway have successfully been overexpressed, e.g., anthranilate synthase (AS), 1-deoxy-d-xylulose synthase (DXS), TDC, STR, G10H or G8O, and apoplastic peroxidase in *C. roseus* cells (Canel et al. 1998), hairy roots (Peebles et al. 2010; Wang et al. 2010; Jaggi et al. 2011), and plants (Pan et al. 2012). In addition, overexpression of the transcription factors ORCA3 in *C. roseus* resulted in elevated levels of some TIA (van der Fits and Memelink 2000; Wang et al. 2010; Pan et al. 2012). However, the precursors from primary metabolism seem to be the limiting factors for increasing production. Therefore, channeling of the metabolic flux towards TIA biosynthesis might be an important target for metabolic engineering to improve TIA production and to reduce the production costs with plants or plant cell cultures.

Several key biosynthesis enzymes in the TIA pathway have been characterized and overexpressed in *C. roseus*. The enzyme that catalyzes the conversion of GPP into geraniol, i.e. geraniol synthase (CrGES) has been isolated and characterized from *C. roseus* (Simkin et al. 2013). This plastidial localized-enzyme is of interest since geraniol is considered as the limiting upstream precursor in TIA biosynthesis; feeding geraniol increased mono-TIA production in *C. roseus* hairy roots (Morgan and Shanks 2000), cell suspension cultures (Lee-Parsons and Royce 2006), and leaves (Kumar et al. 2015).

In the present study, we overexpressed plastidial *CrGES* gene in a non-TIA producing *C. roseus* cell line to increase TIA production in the cell line. Moreover, the *CrGES* gene was also expressed in the cytosol by removing its plastidial leader peptide, to make it available for GPP pool in cytosol. It is conceivable that through *CrGES* gene expression in the cytosol may overcome a possible limitation in transport of geraniol from plastid to the cytosol, thus the mevalonate pathway can be directly linked to the TIA pathway and possibly lead to increased TIA biosynthesis by feeding mevalonic acid. The levels of targeted (TIA and TIA precursors) and non-targeted metabolites were analyzed to determine changes in metabolic profiles of the transformed *C. roseus* cell suspension cultures.

## Materials and methods

### Cell culture

*Catharanthus roseus* cell suspension cultures (cell-line MP183L) were subcultured weekly by transferring 10 ml culture into 50 ml of Linsmaier and Skoog (LS) medium (Linsmaier and Skoog 1965) containing 30 g/l sucrose, 2 mg/l NAA, and 0.2 mg/l kinetin. The cultures were grown on a gyratory shaker at 120 rpm at 25 °C in 16/8 h light/dark regime (20 µE/m^2^/s) at 70% relative humidity.

### Cloning, vector constructions and transformation

The *geraniol synthase* gene of *Catharanthus roseu*s (*CrGES*) consists of a 1770 bp DNA sequence that encodes a protein of 589 amino acids in length. Geraniol synthase is localized in plastids as suggested by the leader peptide encoded in its cDNA (Simkin et al. 2013). To overexpress *CrGES* in the plastids or in the cytosol, a full-length *CrGES* fragment or a truncated version of *CrGES* lacking the first 156 coding nucleotides (Δpl*CrGES*) were constructed. Primers were designed for different vectors employed for different purposes, i.e., pRT101 for constitutive expression and pTH2-Δ*EcoRI* containing sGFP for protein localization studies. The pTH2-Δ*EcoRI* plasmid used in this study is a derivative of pTH2 (Niwa 2003) by adding the “EKE” linker in the *EcoRI* site, resulting in loss of the *EcoRI* site and introduction of a *KpnI* site. In order to insert *CrGES* or Δpl*CrGES* fragments between the CaMV 35S promoter and sGFP in the pTH2-Δ*EcoRI*, a *SalI* site was introduced at the 5′ and 3′ of the inserts. For constitutive expression constructs, *CrGES* and Δpl*CrGES* fragments were amplified with a *SalI* site at the 5′ and *XbaI* site at the 3′, which is compatible with *XhoI* and *XbaI* sites in the pRT101 plasmid.

For transient expression, and constitutive constructs; the forward primers for *CrGE*S and Δpl*CrGES* were 5′-GTCGACAAAATGGCAGCCACAATTAGTAACC-3′ and 5′-GTCGACAAAATG TCTCTGCCTTTGGCAACT-3′, respectively. The reverse primer for the transient expression study was 5′-GTCGACAAAACAAGGTGTAAAAAACAAAGC-3′; while for constitutive constructs, the reverse primer was 5′-TCTAGATTAAAAACAAGGTGTA AAAAACAAAGC-3′ (Supplement 1). Fragments were amplified by PCR (MyCycler Thermal Cycler, Biorad) with following procedures: 98 °C, 1 min; 35 cycles, 98 °C, 15 s; 57 °C, 20 s; 72 °C, 1 min; 72 °C, 5 min. The PCR products were cloned into a pJET1.2/blunt cloning vector (Thermo Scientific, Pittsburgh, PA, USA) and sequenced for confirmation. Subsequently, the verified *CrGES* and Δpl*CrGES* fragments were ligated into pTH2-Δ*EcoRI* plasmid excised with *SalI* (transient expression construct, Niwa 2003) and pRT101 plasmid excised with *XhoI* and *XbaI* (constitutive construct, Töpfer et al. 1987) (Supplement 2).

Plasmids containing *CrGES* and Δpl*CrGES* were introduced into *C. roseus* cells via biolistic transformation (van der Fits and Memelink 1997). The control cells were transformed with the corresponding plasmid without insert. For subcellular localization studies, transformed cells with transient expression constructs were placed on solid LS medium and viewed after 24 h using a Zeiss Observer laser scanning microscope equipped with fluorescence filters. The transformed cells with constitutive expression constructs were cultured on solid LS medium containing 50 µg/ml hygromycin. The individual putative transformed calli grown on this selective medium were converted to cell suspensions and subcultured every week by transferring 10 ml of cell suspension into 50 ml LS medium containing 30 g/l sucrose, 2 mg/l NAA, 0.2 mg/l kinetin, and 50 mg/l hygromycin.

The established cell suspension cultures (after 3 passages) were harvested at day 7 after subculturing. Harvested cells were washed three times with deionized water and frozen in liquid nitrogen. Aliquots of the samples were stored at − 80 °C for RNA extraction and the remaining biomass was lyophilized for 72 h prior to metabolite analysis.

### RNA extraction, northern blot, reverse transcriptase PCR

Frozen cells were ground to a fine powder in liquid nitrogen. Total RNA was extracted with two volumes of hot phenol buffer (1:1 mixture of phenol and 100 mM lithium chloride [LiCl], 10 mM Na-EDTA, 1% SDS, 100 mM Tris) and one volume of chloroform. RNA was precipitated overnight (4 °C) with LiCl at a final concentration of 2 M, washed twice with 70% ethanol, and suspended in water.

Northern blot analysis was performed as described by Memelink et al. (1994) with some modifications. 10 µg RNA samples were subjected to electrophoresis on 1.5% agarose/1% formaldehyde gels, and blotted to GeneScreen nylon membranes (Perkin-Elmer Life Science, Boston, MA, USA). Blots were prehybridized for several hours in 1 M NaCl, 10% dextran sulfate (sodium salt, Sigma), 1% SDS, and 50 µg/ml denatured salmon sperm DNA at 65 °C before addition of denaturated ^32^P-labeled DNA probes. After overnight hybridization, blots were washed twice at 42 °C for 30 min with 0.1 × SSPE (saline/sodium/phosphate/EDTA) and 0.5% SDS. Finally, the blots were washed briefly with 0.1 × SSPE at room temperature. Blots were exposed to X-ray films (Fuji, Tokyo, Japan).

Reverse transcription was carried out using the Revert Aid™ H Minus First Strand cDNA Synthesis Kit (Fermentas) following to the manufacturer’s instruction. The cDNA synthesized from each sample was used as template in PCR. Negative controls were performed by excluding reverse transcriptase enzyme in the reaction.

### Mevalonic acid feeding and jasmonic acid elicitation

The cell suspension cultures were subcultured weekly. Four days after subculturing, mevalonic acid (0.5 µM final concentration) was fed to the *C. roseus* cell suspensions constitutively overexpressing *geraniol synthase* in the cytosol (Δpl*CrGES*). The cells were harvested after 24 h, freeze-dried for 72 h and stored prior to TIA analysis.

For elicitation, jasmonic acid (Sigma) at a final concentration of 100 µM was added to the *C. roseus* cell suspensions constitutively overexpressing *geraniol synthase* in the plastids (*CrGES*) and cytosol (Δpl*CrGES*), and the control culture on the 5th day after subculturing. The elicited cells were harvested after 24, 48, and 72 h. Samples were freeze-dried for 72 h and stored prior to TIA analysis.

### Analysis of terpenoid indole alkaloids

Freeze-dried cells (100 mg) were extracted with 5 ml methanol, vortexed, sonicated for 20 min, and centrifuged for 30 min (3500 rpm). The dried supernatant was suspended in 1 ml phosphoric acid (1 M). Samples were subjected for terpenoid indole alkaloid and precursor analysis using high performance liquid chromatography-diode array detector (HPLC-DAD) (Agilent Techologies Inc., Santa Clara, CA, USA) as described by Saiman et al. (2014).

### Geraniol analysis

Freeze-dried cells (100 mg) were extracted with 5 ml dichloromethane, vortexed, ultrasonicated for 10 min, and centrifuged for 10 min (3000 rpm, 4 °C). The eluent was concentrated under a flow of nitrogen and 1 µl of concentrated extract was injected into gas chromatography-mass spectroscopy (GC–MS) (Agilent Technologies Inc.) equipped with a DB-5 capillary column (30 m × 0.25 mm i.d., film thickness of 0.25 µm) (J&W Scientific Inc., Folsom, CA, USA). The initial oven temperature was 45 °C for 1 min, and was increased to 300 °C at a rate of 10 °C/min and held for 5 min at 300 °C. Geraniol standard compound (Sigma) was used for identification.

### NMR and multivariate data analysis

Freeze-dried cells (25 mg) were extracted with a ratio 1:1 of CD_3_OD and KH_2_PO_4_ buffer. The latter was prepared in D_2_O, adjusted pH to 6.0, and added 0.01% (w/w) trimethylsilyl propanoic acid (TMSP) as internal standard. The mixture was vortexed for 10 s, sonicated for 10 min, and centrifuged for 15 min (14,000 rpm). Samples were analyzed using 500 MHz NMR (Bruker, Karlsruhe, Germany). NMR spectra were manually phased, baseline corrected, and calibrated to TMSP resonance at 0.0 ppm using XWIN NMR version 3.5 (Bruker). AMIX software (Bruker) was used for bucketing (width *δ* 0.04) and data reduction of the ^1^H-NMR spectra (*δ* 0.40–10.00) using total intensity scaling. Multivariate data analysis was performed with the SIMCA software version 12.0 (Umetrics, Umeå, Sweden). Analysis of variance (ANOVA) followed by Duncan’s Multiple Range Test (DMRT) was performed on IBM SPSS Statistics 20 (SPSS Inc., Chicago, IL, USA) to determine statistical differences (*P* < 0.05) between means of groups.

## Results and discussion

### GES cloning and cell transformation

A full fragment of *C. roseu*s’s *geraniol synthase* (*CrGES*, Genbank ID: JN882024, Simkin et al. 2013) and a truncated version of Δpl*CrGES* without the plastidial leader peptide, i.e. lacking the first 156 coding nucleotides, were produced by a PCR-based strategy. The PCR products have shown the expected size of *CrGES* and Δpl*CrGES* fragments and their identities have further been confirmed by sequencing and restriction enzyme analysis (Supplement 3). *CrGES* or Δpl*CrGES* was constructed into the aforementioned plasmid vectors and transferred into *C. roseus* cells by particle bombardment. For the constitutive expression construct using pRT101 vector, an average of 10 putatively transformed calli were obtained per plate of 50 mm diameter size.

### Subcellular localization study

**Fig. 2:**
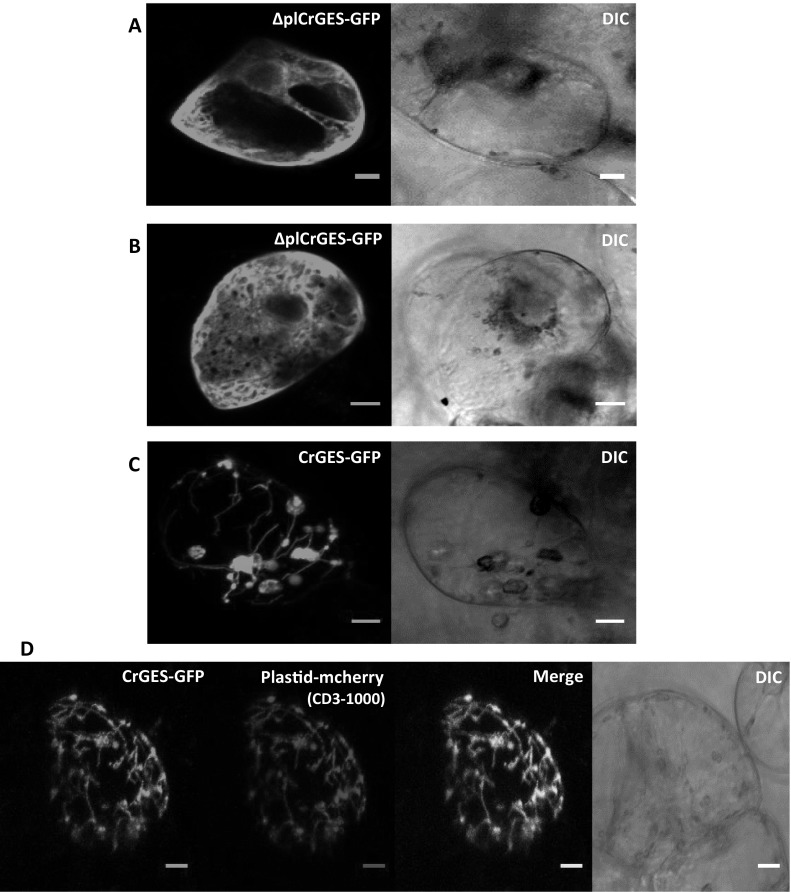
Subcellular localization of Δpl*CrGES*-GFP (**a, b**) and *CrGES*-GFP **c** in cytosol and plastid/stromules of *Catharanthus roseus* cells, respectively. Co-localization of the two fluorescence signals appeared in yellow when merging the two individual (green/red) color images **d**. The cell morphology is observed with differential interference contrast (DIC) microscopy. Bars correspond to 10 µm. (Color figure online)

To confirm the subcellular localization of *CrGES* and Δpl*CrGES*, the expression of the construct pTH2-Δ*EcoRI::CrGES*-GFP and pTH2-Δ*EcoRI*::Δpl*CrGES*-GFP transformed into *C. roseus* cells was analyzed. The results show that the truncated *CrGES* without plastidial leader peptide fused with GFP (Δpl*CrGES*-GFP) was displaying fluorescence in the cytosol (Fig. 2a, b). In accordance to Simkin et al. (2013), the full length *CrGES*-GFP fusion protein signal was located in the plastid stroma and stromules (Fig. 2c). Co-bombardment of *CrGES*-GFP and plastid-mCherry marker (Nelson et al. 2007) in the *C. roseus* cells also show that the fluorescence signal of *CrGES*-GFP matched with those of the plastidial marker (Fig. 2d), thus confirming its localization in the plastid stroma and stromules. By removing its plastidial leader peptide, the enzyme was expressed in the cytosol.

### Gene expression

Expression of *CrGES* and Δpl*CrGES* in the putatively transformed *C. roseus* cells was analyzed using Northern blots and reverse transcriptase PCR (Supplement 4). Northern blot analysis showed expression of *GES* in some of the putatively transformed cell lines. The reverse transcriptase PCR also showed *GES* expression and expected band sizes of the *CrGES* or Δpl*CrGES* fragments in the positive transformed cell lines. Therefore, these results confirm the overexpression of the *CrGES* (plastid) and Δpl*CrGES* (cytosol) in some of the transformed *C. roseus* cell suspensions cultures.

### Terpenoid indole alkaloid analysis

The overexpression lines (*CrGES* and Δpl*CrGES*) and control lines (transformed with empty vector) of *C. roseus* cells were analyzed for the levels of TIA, iridoid and indole precursors using an HPLC-DAD. Neither TIA nor iridoids were detected in the transformed or control *C. roseus* cell cultures. Only tryptophan and tryptamine were detected in all transformed cell lines, similar to the non-TIA producing *C. roseus* cell-line (MP183L). This result indicates that geraniol synthase overexpression in the plastid or cytosol of *C. roseus* cells does not directly enhance the flux towards iridoids and TIA.

The geraniol synthase gene without its plastidial leader peptide (Δpl*CrGES*) was overexpressed in the *C. roseus* cell cultures to overcome a limitation of geraniol transport from plastids to the cytosol. It is further hypothesized that by feeding mevalonic acid in the Δpl*CrGES* overexpression line, the pool of C5 precursors in mevalonate pathway could be increased, and the presence of geraniol synthase gene in cytosol may open a metabolic link between GPP available in the cytosol or GPP derived from the mevalonate pathway to the iridoids and subsequently increase the production of TIA. However, feeding mevalonic acid to the Δpl*CrGES* overexpression line did not result in production of iridoids or TIA. Despite the additional feeding, the level of free cytosolic GPP in these cells is presumably too low to allow conversion by the cytosolic GES to support detectable changes in metabolite composition towards the iridoids and TIA. Cytosolic GPP might be increased by co-transformation with a cytosolic localized GPPS, thus creating and improving a metabolic bridge between the mevalonate pathway and the iridoid/TIA pathway.

A lack of one or more biosynthesis enzymes upstream or downstream geraniol synthase could be the reason that the transformed *C. roseus* MP183L cell lines (non-producing TIA line) did produce neither TIA nor iridoid precursors in both the Cr*GES* and Δpl*CrGES* lines. Our previous study showed that the non-TIA producing CATL cell line (a derivative of MPL183L line) has lower gene transcript levels of *DXR* (sevenfold), *GPPS* (threefold) and *G8O* (*G10H*; 144-fold) compared to the TIA-accumulating CRPP cell line (Saiman et al. 2014), thus probably also in MP183L cell line which would explain the lack of production. Wang et al. (2010) and Pan et al. (2012) showed that overexpression of *G8O* (*G10H*) in *C. roseus* hairy roots and plants, respectively, increased the levels of several TIA which indicates that the G8O (G10H) availability is critical for TIA production. It is thus important to consider both the metabolic and overall pathway-related gene expression profiles of the GES overexpressing *C. roseus* MP183L cell line when evaluating TIA productivity and to compare such data to a cell line or other system accumulating iridoids and TIA, e.g. the earlier identified TIA producing CRPP line (Saiman et al. 2014). This may give some insight in the role of geraniol synthase activity in the carbon flux towards the iridoids and TIA, and find additional limiting factors in the biosynthesis pathway.

**Fig. 3:**
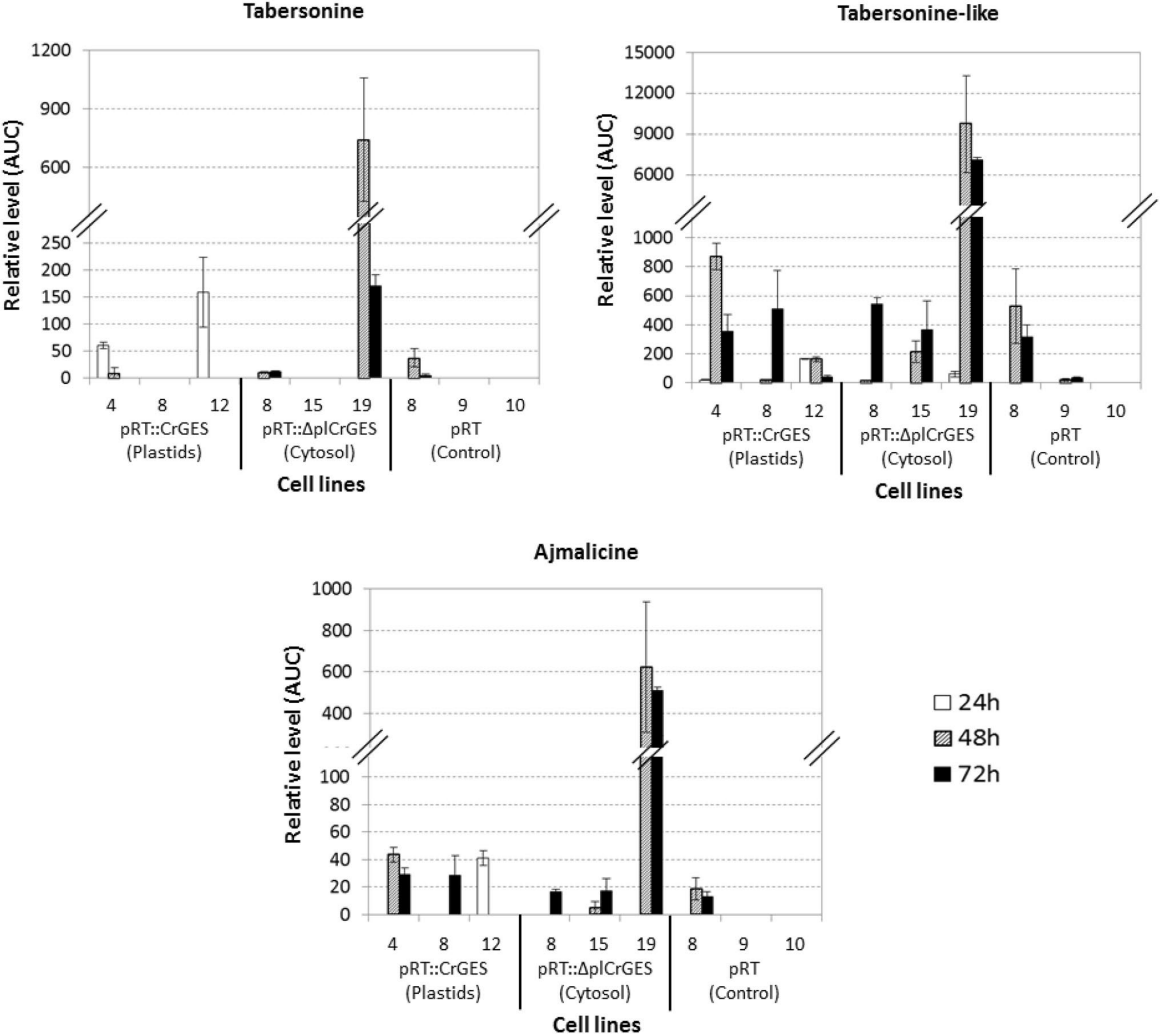
Relative levels of terpenoid indole alkaloids (ajmalicine, tabersonine, and tabersonine-like compound) detected by HPLC-Diode Array Detector (DAD) in jasmonic acid-elicited *Catharanthus roseus* cell suspensions constitutively overexpressing *CrGES* (plastids; cell line 4, 8, and 12), Δpl*CrGES* (cytosol; cell line 8, 15, and 19) and empty vector pRT (control; cell line 8, 9, and 10). All samples were elicited with jasmonic acid (100 µM) at the fifth day after subculturing and harvested at 24, 48, and 72 h after elicitation. Results are the mean of two replicates; error bars indicate the two values. AUC is area under the curve value

In order to further analyze the TIA production in this study, a defense signaling molecule jasmonic acid, which is often used as elicitor to induce plant secondary metabolism, was applied to the cell cultures. Jasmonate has been reported to induce all known TIA pathway genes including *GES* (Simkin et al. 2013), *G8O* or *G10H* (Collu et al. 2001), *ASα, DXS, TDC, STR*, and *SGD* (van der Fits and Memelink 2000), resulting in increased levels of TIA in *C. roseus* cell suspensions (El-Sayed and Verpoorte 2004; Vázquez-Flota et al. 2009). Our previous study also shows that TIA and carotenoid levels were increased upon JA elicitation, whereas the phytosterol levels remained constant, indicating an enhanced availability of precursors through the MEP pathway (Saiman et al. 2015). As expected some TIA, i.e. ajmalicine, tabersonine, and a tabersonine-like compound were accumulated after eliciting the overexpressed and control cell cultures (Fig. 3). Tryptamine levels also increased after elicitation (data not shown), as jasmonic acid induces the expression of *TDC* (van der Fits and Memelink 2000). However, the iridoid precursors were, if present, below detection level, indicating one or more rate-limiting steps in the monoterpenoid biosynthesis in these cell cultures. Most of the cell lines accumulated the alkaloids at 48 and 72 h after elicitation, in which the cell line Δpl*CrGES* #19 accumulated higher levels of alkaloids compared to the other cell lines (Fig. 3). However, the alkaloid production levels in the control lines were quite variable upon elicitation; whereas one empty vector control line did not accumulate alkaloids after elicitation, control line #8 produced alkaloids at a comparable level to that produced by the *CrGES* or Δpl*CrGES* lines. In this perspective only line Δpl*CrGES* #19 revealed a higher TIA production than controls. Therefore, it is confirmed that jasmonic acid induces TIA production in cell-line MP183L, but after elicitation, the variation between GES transformed and control lines is too high to conclude upon a positive effect of the GES transformation.

Gene expression profiling of the control and GES lines might clarify the difference in jasmonate response and production levels (Goklany et al. 2009, 2013). The lack of induced production upon JA elicitation could well be due to a lack of JA sensitivity. In this respect, the jasmonate response can be monitored within minutes to a few hours upon treatment at the level of *JAZ*/*ZIM, MYC* and *ORCA* gene expression as part of the JA responsive transcription factor cascade (Goklany et al. 2013). As the major differences in TIA productivity of control and GES lines relate to ajmalicine and tabersonine production levels, some pathway genes could be selected for gene expression study to support the analytical data on metabolite accumulation. For instance, at least *GES, G10H, TDC* and *STR* expression will already indicate TIA production capacity, while additional monitoring of *GPPS, LAMT, SLS* and some genes involved in the intermediate iridoid steps between 10-hydroxygeraniol and loganic acid (Miettinen et al. 2014) might explain low or high productivity. It is also of interest to consider known repressors of the pathway genes, such as *ZCT1, ZCT2*, and *ZCT3*, as they might still block gene expression and production despite elicitation and feeding (Goklany et al. 2013).

### Geraniol analysis

As overexpressing geraniol synthase in the plastids and cytosol of *C. roseus* cell cultures did not improve production of TIA and iridoids, it was of interest to analyze geraniol; a conversion product of GPP by geraniol synthase. It is known that the *C. roseus* cell line does not accumulate geraniol at normal growth condition. By using GC–MS, geraniol was not detected in the transformed cell lines, neither in the plastidial *CrGES*, nor in the cytosolic Δpl*CrGES* lines. It is presumed that this might be due to the (1) lack of carbon-five precursors because of limiting activity of upstream biosynthesis enzymes, (2) competing pathways acting on GPP, e.g., leading to FPP and sterols or to GGPP and carotenoids, or (3) conversion of geraniol to glycosylated or other derivatives. Dong et al. (2013) reported that tobacco plants overexpressing geraniol synthase from *Valeriana officinalis* (*VoGES*) predominantly accumulated geraniol glycoside. Further work is required to analyze glycosylated geraniol in the transformed *C. roseus* cell lines.

It was reported that expression of *VoGES* to the cytosolic mevalonate compartment resulted in 30% lower geraniol glycoside than the plastidial targeted *VoGES* (Dong et al. 2013). This indicates a smaller pool of GPP in the mevalonate pathway compared to the MEP pathway. It may be interesting to overexpress both *GPPS* and *GES* in the mevalonate pathway of *C. roseus* to improve the formation of iridoids based on mevalonate precursors. Wu et al. (2006) showed that the co-expression of *GPPS* and *limonene synthase* (*LS*) in the cytosolic mevalonate pathway increased production of limonene sixfold compared to the single overexpression of *LS*. However, the targeted subcellular compartment for *GPPS* expression in the mevalonate pathway needs to be evaluated since the IPP isomerase (IDI) and FPPS, which were generally regarded as cytosolic enzymes, were reported to be localized in peroxisomes (Sapir-Mir et al. 2008; Thabet et al. 2011). Nevertheless, Thabet et al. (2011) did not exclude the possibility that a certain proportion of the FPPS is also localized in the cytosol. Furthermore, down-regulation of *FPPS* may also be an interesting approach since a mutated FPPS yeast strain overexpressing *Ocimum basilicum’s geraniol synthase* (*ObGES*) accumulated geraniol produced from the available GPP pool (Fischer et al. 2011).

### NMR-based metabolomics and multivariate data analysis

Although the geraniol synthase overexpressing *C. roseus* cell lines in this study were found not to accumulate TIA or iridoid precursors, other metabolic changes may of interest to be investigated. The cell lines of *CrGES*, Δpl*CrGES*, and control-empty vector were analyzed by NMR-based metabolomics and the data were subjected to multivariate data analysis.

**Fig. 4:**
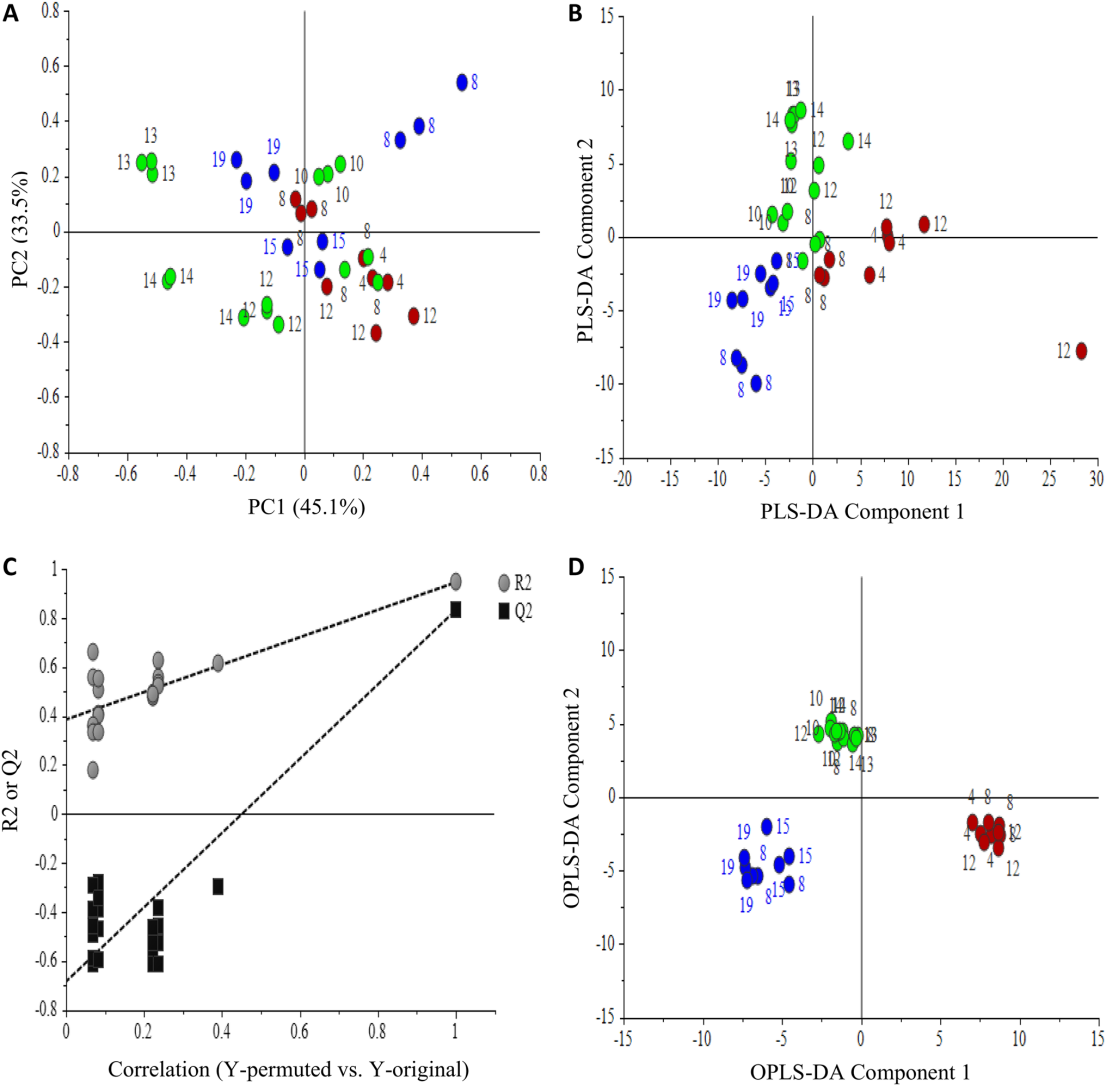
Multivariate data analyses of *Catharanthus roseus* cells constitutively overexpressing *CrGES* (red color), Δpl*CrGES* (blue color), and the control *C. roseus* cells transformed with empty vector (green color). Principal component analysis (PCA) score plot (**a**), partial least squares-discriminant analysis (PLS-DA) score plot (**b**), and orthogonal projection to latent structures-discriminant analysis (OPLS-DA) score plot (**d**). The numbers in the score plots represent the cell-line label. Validation of PLS-DA by permutation test (**c**). R2 is a measurement of the model’s goodness of fit. Q2 is a measurement of the predictive ability of the model. (Color figure online)

Principal component analysis (PCA) which is an unsupervised clustering method shows 45 and 33% variation of PC1 and PC2, respectively (Fig. 4a). However, PCA cannot clearly separate the different cell lines, which means that the variation between the groups is smaller than that within the individual cell lines. Therefore, partial least squares-discriminant analysis (PLS-DA) was applied to the same ^1^H-NMR data to specifically examine the metabolite differences between the three different transformed cell lines. Partial least squares-discriminant analysis (PLS-DA) is a supervised multivariate data analysis that uses information in another matrix. In addition to the X-matrix of NMR data, three groups (*CrGES*, Δpl*CrGES*, and empty vector) were assigned for the Y-matrix in PLS-DA. Figure 4b shows the PLS-DA score plot of the samples, in which the separation between the groups is considerably better. The PLS-DA model was validated by the permutation method through 20 applications (Fig. 4c). Orthogonal projection to latent structures-discriminant analysis (OPLS-DA) was applied to further improve the separation (Fig. 4d). Some major metabolites present in the samples were identified by their NMR signals (Table 1) and different levels in the aromatic region of the ^1^H-NMR spectra can be observed among the samples (Supplement 5).

**Table 1:** ^1^H chemical shift (*δ* in ppm) and coupling constants (*J* in Hz) of some metabolites detected in the transgenic and control cell cultures of *Catharanthus roseus*

Compounds	Chemical shift (ppm) and coupling constant (Hz)
Leucine	*δ* 0.97 (*d, J* = 6.8); *δ* 0.99 (*d, J* = 6.8)
Isoleucine	*δ* 0.96 (*t, J* = 7.5); *δ* 1.03 (*d, J* = 7.0)
Valine	*δ* 1.01 (*d, J* = 7.0); *δ* 1.06 (*d, J* = 7.0)
Threonine	*δ* 1.34 (*d, J* = 6.6)
Alanine	*δ* 1.49 (*d, J* = 7.2)
Glutamic acid	*δ* 2.04 (*m*); *δ* 2.12 (*m*); *δ* 2.39 (*m*)
Glutamine	*δ* 2.13 (*m*); *δ* 2.46 (*m*)
Malic acid	*δ* 2.68 (*dd, J* = 15.4, 3.3); *δ* 4.28 (*dd, J* = 9.5, 3.2)
Aspartic acid	*δ* 2.82 (*dd, J* = 17.0, 8.0); *δ* 2.95 (*dd, J* = 16.8, 4.0); *δ* 3.92 (*dd, J* = 8.4, 4.0)
Sucrose	*δ* 4.18 (*d, J* = 8.6); *δ* 5.41 (*d, J* = 3.8)
Glucose	*δ* 4.58 (*d, J* = 8.0, *β*-form); *δ* 5.19 (*d, J* = 3.8, *α*-form)
Fumaric acid	*δ* 6.52 (*s*)
Tyrosine	*δ* 6.85 (*d, J* = 8.5) *δ* 7.19 (*d, J* = 8.5)
Tryptophan	*δ* 7.14 (*t, J* = 7.5); *δ* 7.22 (*t, J* = 7.5); *δ* 7.29 (*s*); *δ* 7.48 (*d, J* = 8.0); *δ* 7.73 (*d, J* = 8.0)
Tryptamine	*δ* 7.14 (*t, J* = 7.5); *δ* 7.22 (*t, J* = 7.5); *δ* 7.28 (*s*); *δ* 7.48 (*d, J* = 8.0); *δ* 7.65 (*d, J* = 8.0)
Phenylalanine	*δ* 7.36 (*m*)
Formic acid	*δ* 8.48 (*s*)

*s* singlet, *d* doublet, *dd* double doublet, *t* triplet, *m* multiplet

**Fig. 5:**
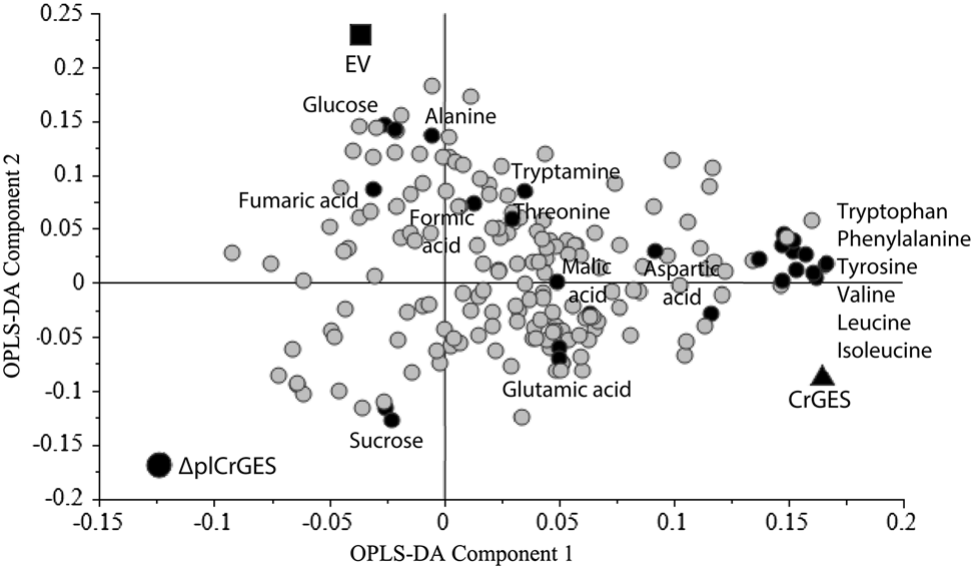
Loading plot of orthogonal projection to latent structures-discriminant analysis (OPLS-DA) of *Catharanthus roseus* cells constitutively overexpressing *CrGES*, Δpl*CrGES*, and the control *C. roseus* cells transformed with empty vector (EV). Variables X (metabolite signals) located closely to variable Y (*CrGES*, Δpl*CrGES*, EV) contributes to the separation of the samples. The grey circles are the metabolites signals and the dark black circles are the signals of identified metabolites listed in the Table 1

**Fig. 6:**
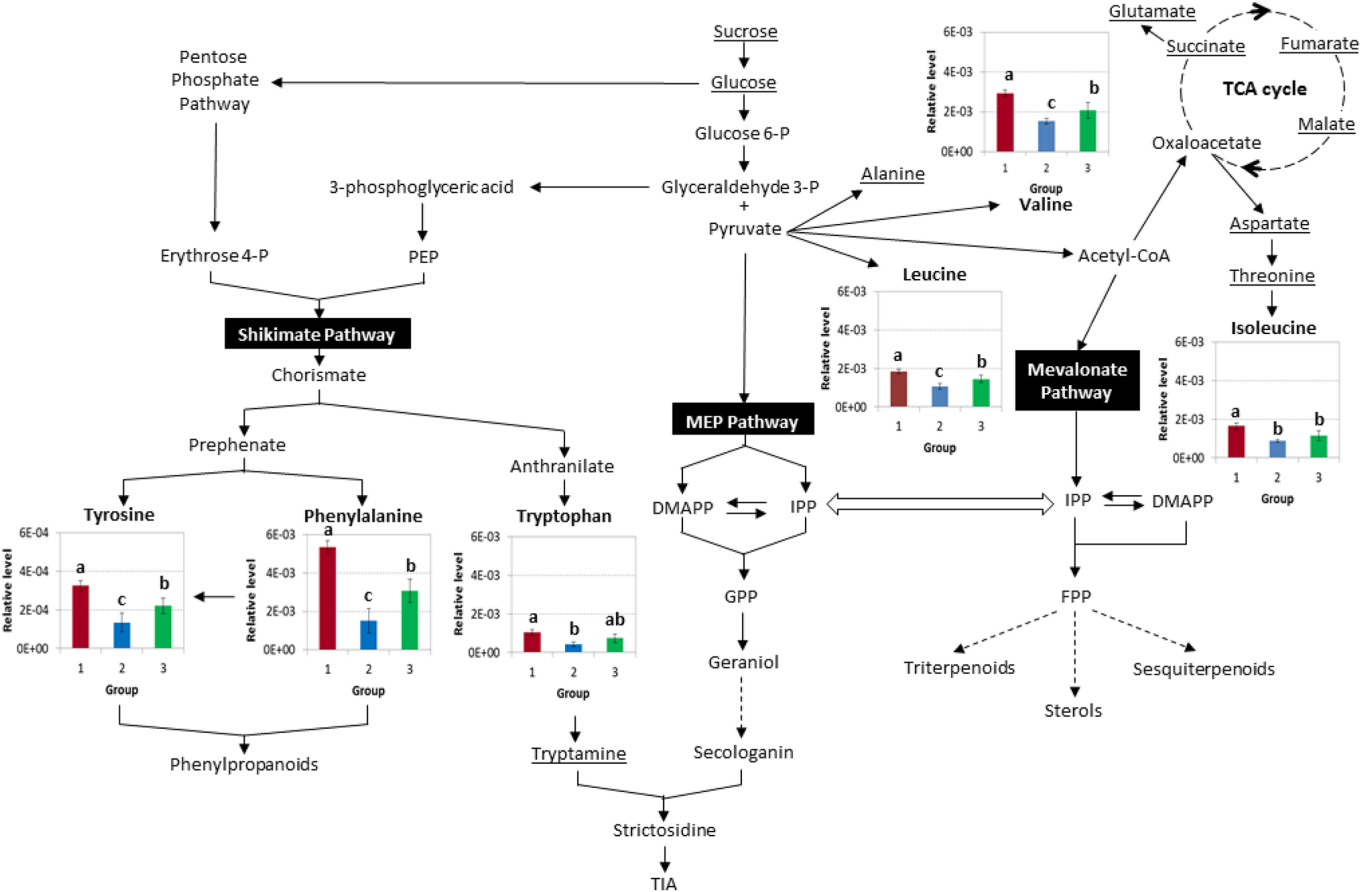
Schematic representation of metabolic differences between *Catharanthus roseus* cell lines overexpressing *CrGES* in the plastids (*1*), Δpl*CrGES* in cytosol (*2*), and empty vector/control (*3*). Relative level of metabolites is the mean area of ^1^H-NMR resonance peak associated to that metabolite. Each group consists of three to five different cell lines and each cell line is represented by three biological replicates. Means ± SD with different letters are significantly different (*P* < 0.05) using ANOVA followed by Duncan’s multiple range test (DMRT). Underlined metabolites are not significantly different in levels

The loadings plot reveals which of these metabolites contribute to the separation of the groups (Fig. 5). In order to confirm that the metabolite are statistically significant for the separation of the groups, an ANOVA test (*P* < 0.05) was performed by comparing the mean value of the relative levels of metabolites between the groups of observation. The result suggests that valine, leucine, isoleucine, tryptophan, phenylalanine, and tyrosine are the metabolites that significantly differ (*P* < 0.05) between the groups (Fig. 6). All of these metabolites were found about twofold higher (1.7–2.5-fold) in plastidial-*CrGES* overexpressing lines compared to the cytosolic-Δpl*CrGES* lines. Except tryptophan, these metabolites were also significantly higher (1.3–1.7-fold) in plastidial-*CrGES* overexpressing lines compared to the control (empty vector) cell lines. These results indicate that overexpressing *CrGES* in the plastids increased the levels of some primary metabolites including aromatic amino acids phenylalanine and tyrosine which derive from the shikimate pathway. Tyrosine and phenylalanine are essential components for plant growth and development. The latter is precursor for lignin, the component of plant cell wall. In addition, phenylalanine-derived compounds play diverse roles in plant adaptation such as plant defense, UV protection, and reproduction. The reason of the increase of metabolism in shikimate pathway after overexpressing plastidial geraniol synthase is not clear but could be related to the localization of shikimate pathway in the plastids. A study by Sung et al. (2011) revealed the enzyme geraniol 8-oxidase (also known as 10-hydroxylase) which catalyze the conversion of geraniol to 8-hydroxygeraniol has dual function in terpenoid and flavonoid/phenylpropanoid pathways. On the contrary, overexpression of Δpl*CrGES* in cytosol showed a reverse effect, in which tyrosine, phenylalanine, leucine, and valine were significantly decreased (*P* < 0.05) compared to the control and the *CrGES* overexpressing cell lines. This indicates that the different subcellular localization of geraniol synthase results in different metabolic effects, thus a suitable targeted compartment is crucial to determine the production of the compounds of interest. Alteration of unrelated biosynthetic pathways may occur upon genetic modification of the targeted pathway. For example, Nagel et al. (2014) showed that overexpression of isoprenyl diphosphate synthase in a conifer plant did not increase the targeted monoterpene and diterpene pathway, instead the flux was diverted to increase the accumulation of geranylgeranyl fatty acid ester that function in plant defense. Such indirect, unrelated metabolic effects remain unnoticed when only applying targeted analysis of pathway metabolites. Only by including untargeted metabolomics approaches, e.g. as possible by NMR spectroscopy, such unexpected roles of introduced genes will be revealed. Further studies are required for a better understanding of the total metabolic network and the roles played by geraniol synthase dependent on its localization.

## Conclusion

In this study, we describe the development of *C. roseus* cell lines overexpressing *geraniol synthase* in the plastid (*CrGES*) or the cytosol (Δpl*CrGES*). The study confirmed the expression of *CrGES* and Δpl*CrGES* in both subcellular compartments. Its product, geraniol was not detected in the transformed cells or present below the detection limit, which either indicates low production and accumulation of geraniol, or a rapid metabolization. Further analyses are needed to reveal if the GES product is formed and subsequently derivatized to glycosylated geraniol or other products. No accumulation of TIA or iridoid pathway precursors was detected in the *C. roseus* cell suspension cultures overexpressing *geraniol synthase*. Addition of mevalonic acid as precursor did not directly stimulate TIA production in the Δpl*CrGES* transformed cell lines; presumably the level of free cytosolic GPP in these cells is too low to support detectable changes in metabolite composition. Co-transformation with a cytosolic localized GPPS may increase the GPP availability in cytosol, thus improving a metabolic link between the mevalonate pathway and the iridoid/TIA pathway. Jasmonic acid elicitation stimulated the TIA production in most transformed cell-lines, but did not cause clear differences in TIA production between overexpression and control cultures. NMR-based metabolomics combined with multivariate data analysis revealed alteration of some primary metabolites in both plastidial and cytosolic *CrGES* overexpressing *C. roseus* cells. In contrast to a higher level of several metabolites of which some are associated to shikimate pathway in the constitutive *CrGES* overexpressing *C. roseus* cells, a lower level of these metabolites was detected in the Δpl*CrGES* overexpressing cultures compared to the control, thus suggesting different metabolic effects related to the subcellular compartmentation of geraniol synthase.

## Electronic supplementary material

Below is the link to the electronic supplementary material.

Supplementary material 1 (PDF 275 KB)

Supplementary material 2 (PDF 191 KB)

Supplementary material 3 (PDF 189 KB)

Supplementary material 4 (PDF 331 KB)

Supplementary material 5 (PDF 301 KB)
