# Supplementary material for: Metabolic alteration of Catharanthus roseus cell suspension cultures overexpressing geraniol synthase in the plastids or cytosol
Source: Plant Cell Tissue Organ Cult. 2018 Feb 24;134(1):41–53. doi: 10.1007/s11240-018-1398-5 (PMC6445406; doi:10.1007/s11240-018-1398-5)
Supplement: Supplementary file 1 — Supplementary material 1 (PDF 275 KB) [file 11240_2018_1398_MOESM1_ESM.pdf]

Supplement 1 The primer sequences used for designing full-length cDNA of *Catharanthus roseus* geraniol synthase (*CrGES*) or a truncated version without its plastidial leader peptide (Δpl*CrGES*). Construct no. 1 and 2 are the inserts for constitutive expression. Construct no. 3 and 4 are the inserts for transient expression.

No	Constructs	Primer sequences	
		Forward	Reverse
1.	*SalI*-CrGES-*XbaI*	5’-GTCGACAAAATGGCAGCCACAATTAGTAACC-3’	5’-TCTAGATTAAAAACAAGGTGTAAAAAACAAAGC-3’
2.	*SalI*-ΔplCrGES-*XbaI*	5’-GTCGACAAAATGTCTCTGCCTTTGGCAACT-3’	5’-TCTAGATTAAAAACAAGGTGTAAAAAACAAAGC-3’
3.	*SalI*-CrGES-*SalI*	5’-GTCGACAAAATGGCAGCCACAATTAGTAACC-3’	5’-GTCGACAAAACAAGGTGTAAAAAACAAAGC-3’
4.	*SalI*-ΔplCrGES-*SalI*	5’-GTCGACAAAATGTCTCTGCCTTTGGCAACT-3’	5’-GTCGACAAAACAAGGTGTAAAAAACAAAGC-3’
