# Supplementary material for: Metabolic alteration of Catharanthus roseus cell suspension cultures overexpressing geraniol synthase in the plastids or cytosol
Source: Plant Cell Tissue Organ Cult. 2018 Feb 24;134(1):41–53. doi: 10.1007/s11240-018-1398-5 (PMC6445406; doi:10.1007/s11240-018-1398-5)
Supplement: Supplementary file 2 — Supplementary material 2 (PDF 191 KB) [file 11240_2018_1398_MOESM2_ESM.pdf]

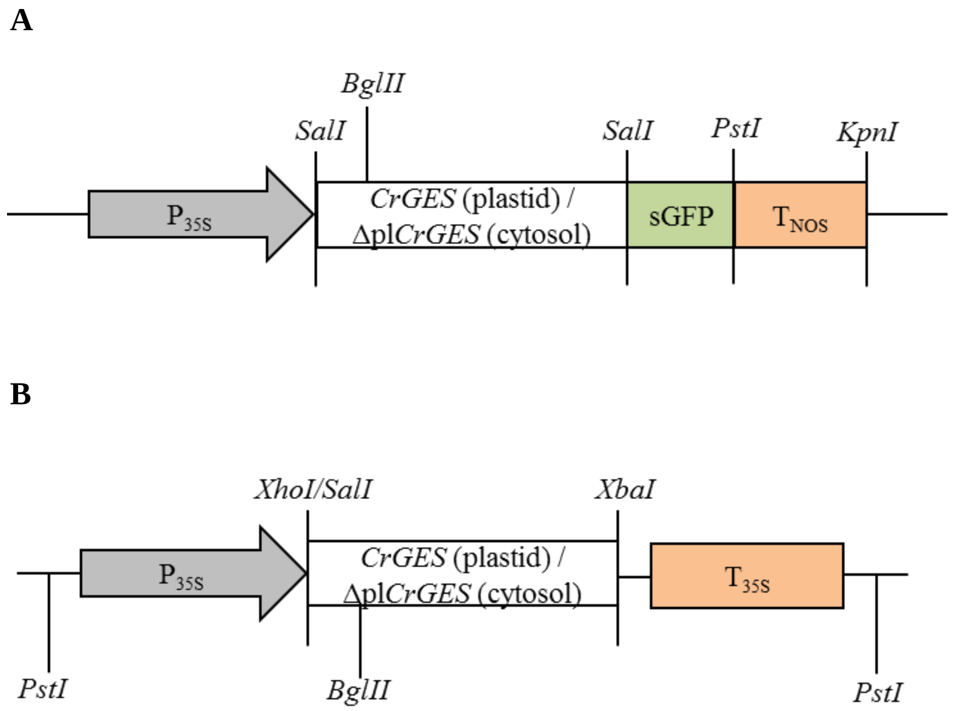


**Supplement 2** Schematic representation of the constructs A) pTH2-Δ*EcoRI* (Niwa 2003) and B) pRT101 (Töpfer et al. 1987) vectors containing either the full-length fragment of *Catharanthus roseus* geraniol synthase (*CrGES*) or the truncated *CrGES* without plastidial leader peptide (Δpl*CrGES*) that are targeting the enzyme to the plastid or the cytosol, respectively. $P_{35S}$: cauliflower mosaic virus 35S promoter, sGFP: synthetic green fluorescence protein (S65T), $T_{NOS}$: nopaline synthase terminator, $T_{35S}$: cauliflower mosaic virus 35S terminator.
