# Supplementary material for: Metabolic alteration of Catharanthus roseus cell suspension cultures overexpressing geraniol synthase in the plastids or cytosol
Source: Plant Cell Tissue Organ Cult. 2018 Feb 24;134(1):41–53. doi: 10.1007/s11240-018-1398-5 (PMC6445406; doi:10.1007/s11240-018-1398-5)
Supplement: Supplementary file 3 — Supplementary material 3 (PDF 189 KB) [file 11240_2018_1398_MOESM3_ESM.pdf]

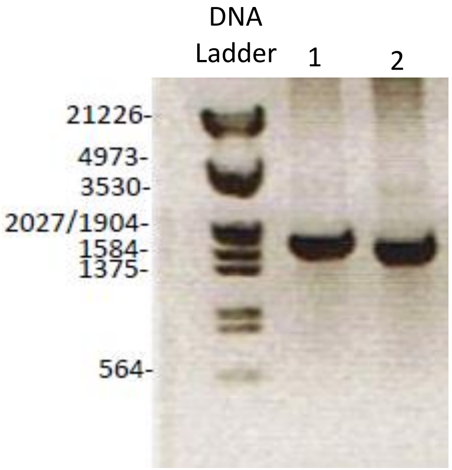

Supplement 3 The PCR products cut with restriction enzymes *SalI* and *XbaI* examined by gel electrophoresis. 1) full-length fragment of *Catharanthus roseus* geraniol synthase (*CrGES*; about 1.8 kb). 2) fragment encoding truncated CrGES without plastidial leader peptide (Δpl*CrGES*; about 1.6 kb).
