# Supplementary material for: Metabolic alteration of Catharanthus roseus cell suspension cultures overexpressing geraniol synthase in the plastids or cytosol
Source: Plant Cell Tissue Organ Cult. 2018 Feb 24;134(1):41–53. doi: 10.1007/s11240-018-1398-5 (PMC6445406; doi:10.1007/s11240-018-1398-5)
Supplement: Supplementary file 4 — Supplementary material 4 (PDF 331 KB) [file 11240_2018_1398_MOESM4_ESM.pdf]

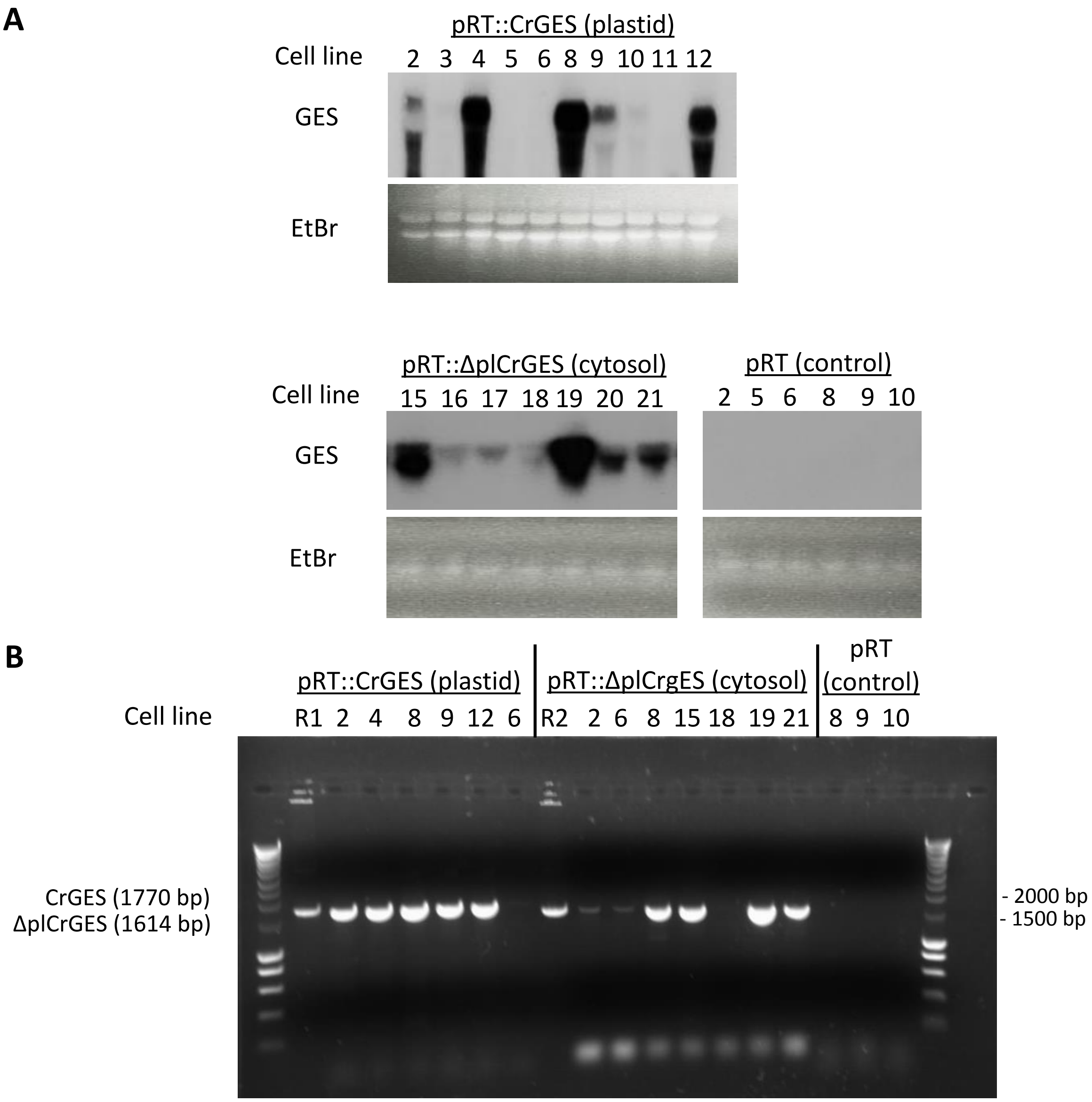

Supplement 4 Northern blot (A) and reverse transcriptase PCR (B) analysis of some transgenic *Catharanthus roseus* cell lines overexpressing *CrGES* (plastid) and Δpl*CrGES* (cytosol). The ethidium bromide stained gel (EtBr) in Northern blot is shown as a control for RNA loading. R1 and R2 is the *CrGES* and Δpl*CrGES* fragments, respectively, which are used as reference for comparing the sizes of the bands
