# Supplementary material for: Metabolic alteration of Catharanthus roseus cell suspension cultures overexpressing geraniol synthase in the plastids or cytosol
Source: Plant Cell Tissue Organ Cult. 2018 Feb 24;134(1):41–53. doi: 10.1007/s11240-018-1398-5 (PMC6445406; doi:10.1007/s11240-018-1398-5)
Supplement: Supplementary file 5 — Supplementary material 5 (PDF 301 KB) [file 11240_2018_1398_MOESM5_ESM.pdf]

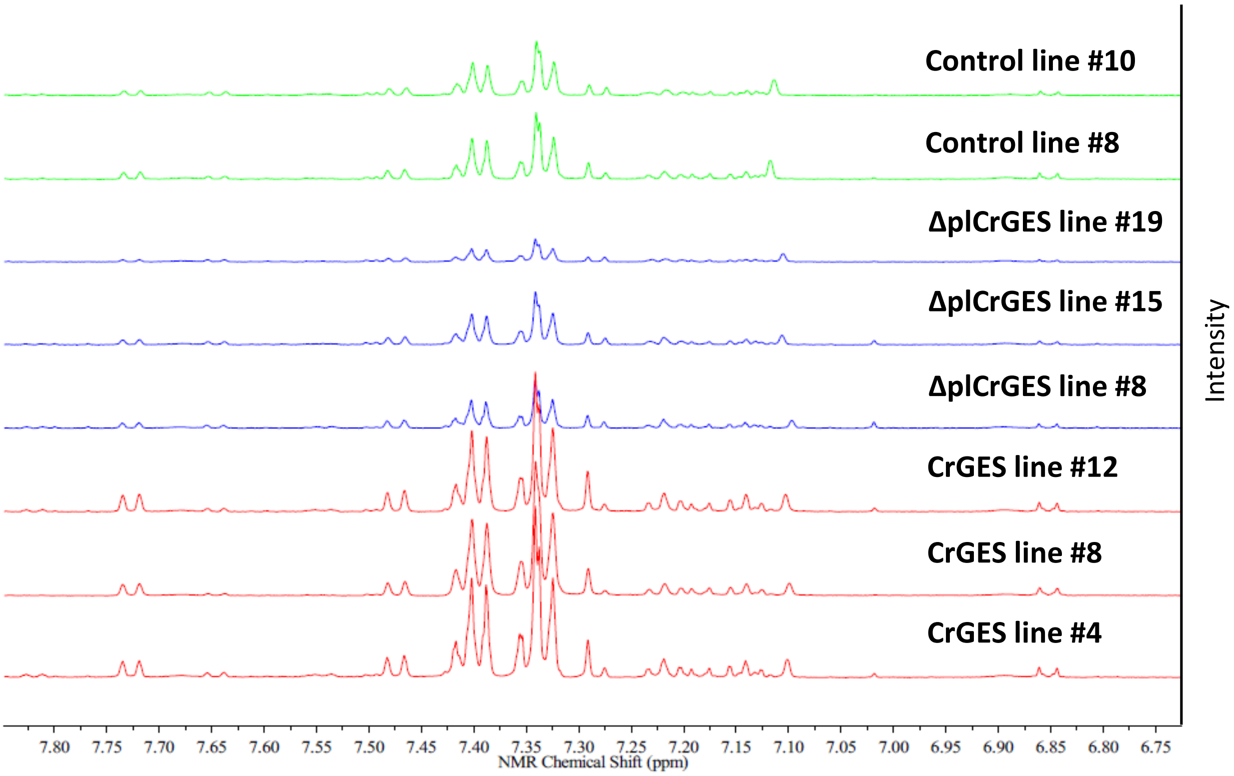

Supplement 5 Comparison of ^{1}H-NMR spectra of aromatic region of two *Catharanthus roseus* control lines (transformed with empty vector) versus different cell lines constitutively expressing *CrGES* and Δpl*CrGES*.
